# Endovascular treatment strategy and clinical outcome of tentorial dural arteriovenous fistula

**DOI:** 10.3389/fneur.2023.1315813

**Published:** 2024-02-02

**Authors:** Guangjian Zhang, Weiwei Zhang, Hanxiao Chang, Yuqi Shen, Chencheng Ma, Lei Mao, Zheng Li, Hua Lu

**Affiliations:** ^1^Department of Emergency, Nanjing Drum Tower Hospital, The Affiliated Hospital of Nanjing University Medical School, Nanjing, China; ^2^Department of Ophthalmology, Third Medical Center of Chinese PLA General Hospital, Beijing, China; ^3^Department of Neurosurgery, Jiangsu Province Hospital, Nanjing, China; ^4^Department of Neurosurgery, The First Affiliated Hospital of Nanjing Medical University, Nanjing, China

**Keywords:** arteriovenous fistulas, endovascular treatment, strategy, outcome, tentorial

## Abstract

**Introduction:**

To evaluate treatment strategies and clinical outcomes following endovascular embolization of tentorial dural arteriovenous fistulas.

**Methods:**

We retrospectively analyzed 19 patients with tentorial dural arteriovenous fistulas admitted to the Department of Neurosurgery at Jiangsu Provincial People’s Hospital between October 2015 and May 2022, all treated with endovascular therapy. To collect and analyze patients’ clinical presentation, imaging data, postoperative complications, and prognosis and to analyze the safety and clinical outcomes of endovascular treatment of tentorial dural arteriovenous fistulas.

**Results:**

Imaging cure was achieved in 18 patients, with the arterial route chosen for embolization in 17 patients and the venous route in one patient; one patient received partial embolization. Staged embolization was performed in four patients. At postoperative follow-up of 9–83 months (37.8 ± 21.2), all 19 patients had recovered well (mRS score ≤ 2). Three patients experienced perioperative complications: intraoperative Onyx reflux into the middle cerebral artery in one patient; postoperative permanent limited left visual field loss and deafness in the left ear in one patient; and transient diplopia, vertigo, and decreased pain and temperature sensation of the left limb in one patient, with no abnormalities on post-procedure magnetic resonance examinations. A total of 17 patients completed a postoperative digital subtraction angiography review during follow-up, and one patient had a recurrence of an arteriovenous fistula.

**Conclusion:**

Endovascular treatment of tentorial dural arteriovenous fistulas is safe and effective. Reduction of the Borden or Cognard classification via eliminating cortical venous reflux through multi-staged embolization or combined open surgery is a reasonable goal of treatment where complete obliteration of the fistula is not achievable.

## Introduction

Dural arteriovenous fistula (DAVF) is an intracranial vascular malformation that is mainly characterized by the formation of an abnormal direct connection between the arteries and veins in the dura. It accounts for 10–15% of intracranial vascular malformations commonly found in the transverse sinus-sigmoid sinus area, cavernous sinus area, tentorium of the cerebellum area, and superior sagittal sinus area (1, 2). DAVF usually occurs in people aged 50–70 years and is slightly more common in men than in women (3). The pathophysiology of DAVF is an obstruction caused by inflammation, thrombosis, and stenosis, which leads to congestion and venous hypertension. Subsequent elevation of venous pressure leads to the formation of a fistula connecting the meningeal artery, dural sinus, and cortical vein. Over time, abnormal blood flow shifts and retrogradation can lead to cortical venous reflux (4). Cortical venous drainage puts patients with DAVF at risk of bleeding, nerve damage, and death.

Tentorial dural arteriovenous fistulas are rare and complicated, with complex feeding arteries and draining veins that are relatively difficult to treat, and their optimal therapeutic strategy is controversial (5). Rezende et al. concluded that arterial embolization was a safe and effective option for 45 patients with TDAVF (6). In their study, all TDAVFs were Borden type III, and they concluded that injection of Onyx glue through the posterior branches of the middle meningeal artery has become the treatment of choice for most TDAVFs. This artery has unique characteristics and is considered a favorable treatment even if it is not the main supplier of the fistula. Advances in endovascular neuroradiology, including devices, have led to this modality becoming the main treatment strategy for tentorial DAVFs. The aim of the present study was to compare the safety and efficacy of one-stage versus multi-stage treatment, to assess the impact of pattern of arterial supply on success of treatment.

## Materials and methods

### Study design

We performed a retrospective review of 145 consecutive DAVF patients treated at a single center, the Department of Neurosurgery of the First Affiliated Hospital of Nanjing Medical University between October 2015 and May 2022. A case series of 20 patients (13.8%) with a tentorial DAVF were identified. One patient was excluded from this study because intraoperative imaging data could not be found. Ethical approval was provided by our institutional ethics committee with written consent obtained from all patients included in this study.

### Participants

The inclusion criteria were as follows: (1) all adult patients above age 18, (2) confirmed TDAVF on digital subtraction angiography (DSA), and (3) patients treated with endovascular therapy to embolize the TDAVF. Patients were excluded (1) where the primary mode of treatment was open surgery, (2) if they had hepatic or renal dysfunction that would preclude the use of contrast media, and (3) if they had other cerebrovascular disorders, such as arteriovenous malformation and Moyamoya disease.

### Interventions

All patients were treated under general anesthesia, heparin was administered, and the appropriate route of endovascular embolization was selected based on the DSA results. We used the Seldinger technique to place a 6F femoral artery sheath and characterized the location and angioarchitecture of the DAVF by sequentially administering iodinated contrast into the bilateral internal and external carotid arteries and vertebral arteries via a 5F catheter. Subsequently, a 6F guide catheter was passed into an appropriately selected feeding artery, and a Marathon microcatheter (eV3, United States) was subsequently used to deliver Onyx glue via this embolization channel into the arteriovenous fistula site. This was done by hand under fluoroscope assistance with sequential contrast runs guiding adjustments to the angle of embolization. A final DSA was performed at the end to confirm the extent of embolization.

### Rationale of treatment

The goal of treatment was complete obliteration of the arteriovenous shunt where possible. Incomplete treatment may result in collateral vessel recruitment and an ongoing risk of bleeding. When complete occlusion of the shunt is not feasible or considered high risk, flow reduction or selective disconnection of the cortical venous drainage was considered.

### Selection of arterial route of embolization

We preferentially use the middle meningeal artery as the main embolic route when it is involved in the blood supply of the DAVF. The middle meningeal artery, occipital artery, meningohypophyseal trunk branches, superior cerebellar artery, and posterior inferior cerebellar artery were chosen to embolize the Galenic or Lawton Type I TDAVF; the middle meningeal artery and posterior inferior cerebellar artery were chosen to embolize the straight Sinus or Type II TDAVF; the middle meningeal artery and posterior meningeal artery were chosen to embolize the torcular or Type III TDAVF; the middle meningeal artery, occipital artery, anterior inferior cerebellar artery, and superior cerebellar artery were chosen to embolize the tentorial sinus or Type IV TDAVF; the middle meningeal artery and occipital artery were chosen to embolize the superior petrosal sinus or Type V TDAVF; the occipital artery was chosen to embolize the incisural or Type VI TDAVF.

### Follow-up

Patients were assessed using the modified Rankin Scale (mRS); grades 0–2 were classified as a good outcome and 3–6 as a poor outcome. Cognitive and neurological deficits, puncture site complications, and radiation-related complications were assessed in the postoperative period. A CT scan was performed 1 month post procedure to assess for asymptomatic hemorrhage or infarction. Digital Subtraction angiographic follow-up was performed 3–6 months after embolization and every 1–2 years thereafter.

## Results

### Patient characteristics

A male preponderance was observed, with 15 males and 4 females. The mean age was 50.4 years (range, 26–64). In terms of Lawton’s classification (7), there were six cases of Lawton type I, two cases of type II, three cases of type III, three cases of type IV, four cases of type IV, and one case of type VI. All patients were symptomatic, with headache being the most common complaint [9 patients (9pts)], followed by dizziness (4pts), impaired consciousness (4pts), nausea and vomiting (1 pt), and periorbital bruising (1 pt). Nine patients had cerebral hemorrhage. The clinical characteristics of all patients and tentorial dural arteriovenous fistula are shown in Table 1.

**Table 1:** Clinical characteristics of all patients and the TDAVF.

Patient	Sex	Age	Clinical presentation	Cerebral hemorrhage	Lawton classification	Feeding arteries	Vessels embolized	Embolization degree	mRS score (preoperative, postoperative)
1	Male	61	Headache	Yes	G1	2	MHT	Total obliteration	4, 1
2	Female	43	Headache	No	G2	10	MMA	Total obliteration	1, 1
3	Female	31	Dizzy	No	G1	14	MMA, OA	Partial obliteration	1, 0
4	Female	47	Headache	Yes	G2	4	PICA	Total obliteration	4, 1
5	Male	51	Unconsciousness	Yes	G3	2	MMA	Total obliteration	5, 0
6	Male	60	Headache	No	G3	5	MMA	Total obliteration	1, 0
7	Female	40	Periorbital bruising	No	G4	8	AICA, SCA, OA, MMA	Total obliteration	1, 2
8	Male	55	Unconsciousness	Yes	G5	3	OA, MMA	Total obliteration	5, 1
9	Male	48	Headache	Yes	G5	2	Transvenous-superior petrosal sinus route	Total obliteration	3, 0
10	Male	52	Headache	No	G6	4	OA	Total obliteration	1, 0
11	Male	59	Headache	Yes	G3	3	PMA	Total obliteration	5, 1
12	Male	64	Unconsciousness	Yes	G1	6	SCA	Total obliteration	5, 0
13	Male	59	Unconsciousness	No	G1	6	MMA	Total obliteration	1, 1
14	Male	56	Headache	No	G1	2	OA, MMA	Total obliteration	1, 0
15	Male	60	Dizzy	No	G4	2	MMA	Total obliteration	3, 1
16	Male	50	Dizzy	No	G5	3	MMA	Total obliteration	1, 0
17	Male	45	Headache	Yes	G1	1	PICA	Total obliteration	3, 1
18	Male	26	Nausea and vomiting	Yes	G5	4	MMA, OA	Total obliteration	4, 1
19	Male	50	Dizzy	No	G4	3	MMA, OA	Total obliteration	1, 1

MHT, meningohypophyseal trunk branches; MMA, middle meningeal artery; OA, occipital artery; AICA, anterior inferior cerebellar artery; SCA, superior cerebellar artery; PICA, posterior inferior cerebellar artery; PCA, posterior cerebral artery. PMA, posterior meningeal artery.

### Treatment outcome

The arterial route was utilized for embolization in 95% (18/19) of cases, with the venous route used for embolization in one case. Complete obliteration of the fistula was achieved in 79% (15pts) via the arterial route in a single setting, 16% (3pts) underwent complete obliteration after staged embolization, partial embolization was performed in one patient (5%) to eliminate the cortical venous reflux aimed at reducing hemorrhage risk. All patients were embolized with Onyx glue and the duration of treatment was 80–175 min (119.5 ± 28.4). The middle meningeal artery was the chosen vessel for embolization in 12 of 13 cases in which the artery was involved in the DAVF, followed by the occipital artery.

### Outcomes and follow-up

After a median follow-up of 9–83 months (37.8 ± 21.2), 18 patients had no neurological deficits, presenting symptoms had resolved in 15 patients, there was no change in three patients, and one patient’s symptoms were worse. Angiographic follow-up with DSA showed lasting obliteration of DAVF in 17 patients but recurrence in one patient, who presented with seizures 1 year after treatment. A radiological cure was achieved after retreatment via the arterial route.

### Complications and adverse events

Three patients experienced post-embolization complications: One patient with a type I tentorial dural arteriovenous fistula, supplied mainly by the meningeal pituitary trunk and posterior cerebral artery and embolized via the meningeal pituitary trunk, had Onyx glue refluxed into the middle cerebral artery on the ipsilateral side. An LVIS stent was used to append the onyx against the arterial wall with no neurological sequelae observed after 8 years of follow-up. A second patient with a type IV fistula suffered a partial visual field defect, left-sided hearing loss, and weakness in both upper limbs (grade 3) following reflux of Onyx glue into the functional vessels. The limb deficits recovered after 4 years of physical rehabilitation, but the other deficits persisted. The third patient with a type V TDAVF with an aneurysm, who underwent embolization via the venous route with coils and Onyx glue, developed transient diplopia, left ptosis, vertigo, dysarthria, left tongue protrusion, reduced pain, and temperature sensation of the left limb. A magnetic resonance examination revealed no abnormalities, and the symptoms resolved after 3 days and were believed to be secondary to vasospasm.

## Discussion

**Figure 1:**
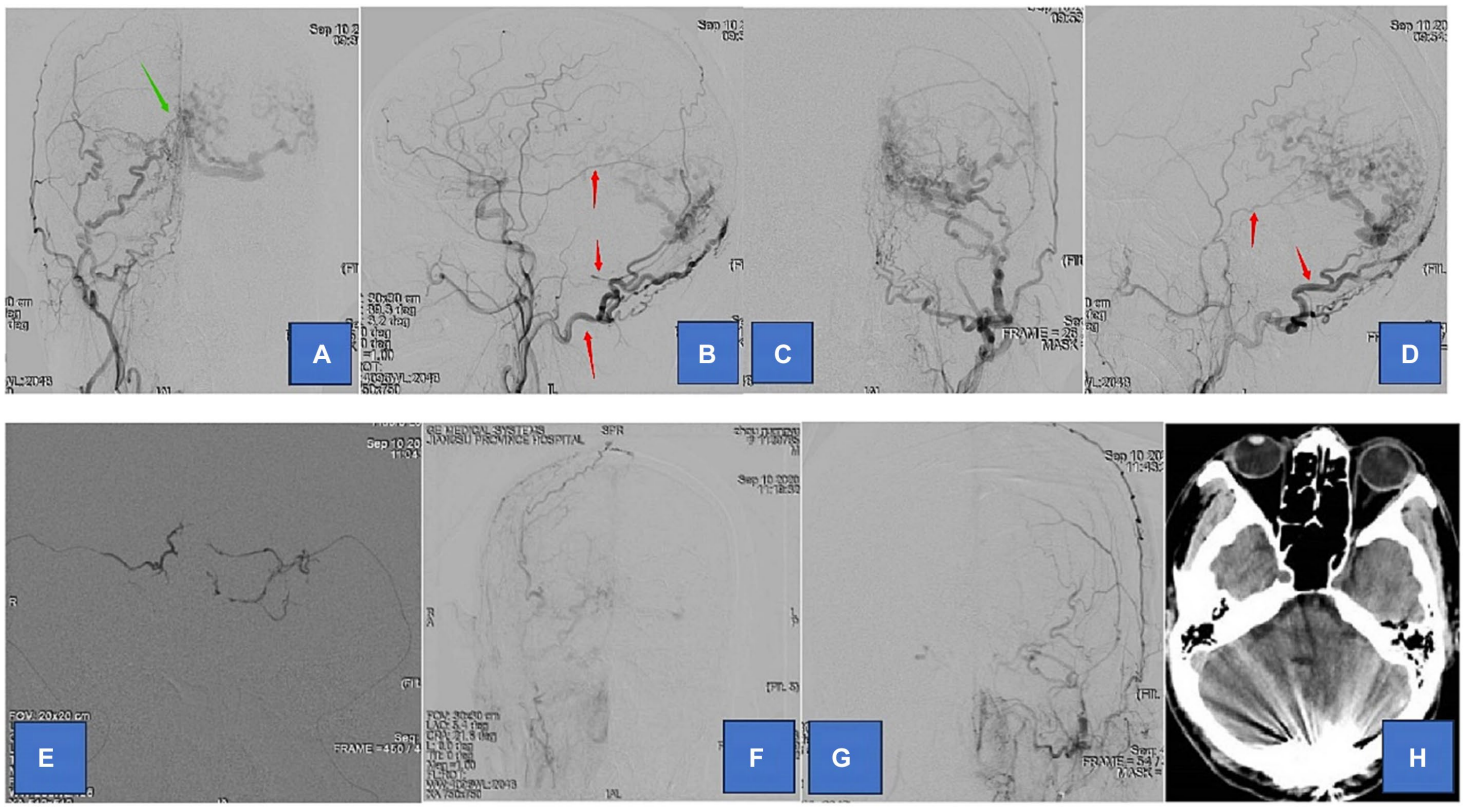
Adult patient presented with headache for 1 month, whose DSA suggested a tentorial dural arteriovenous fistula of type G3. **(A,B)** are the frontal and lateral views of the right external carotid artery, showing the right occipital, middle meningeal, and ascending pharyngeal arteries involved in the blood supply. **(C,D)** are the frontal and lateral views of the left external carotid artery showing the left occipital, middle meningeal, and posterior auricular arteries involved in the blood supply. **(E)** We chose the bilateral middle meningeal artery route for the embolization of TDAVF. **(F,G)** are postoperative bilateral external carotid arteriograms showing complete embolization of the TDAVF. **(H)** is a postoperative cranial CT showing hyperdense embolic shadow in the sinus convergence area. Red arrows point to arteries, and green arrows point to TDAVF.

**Figure 2:**
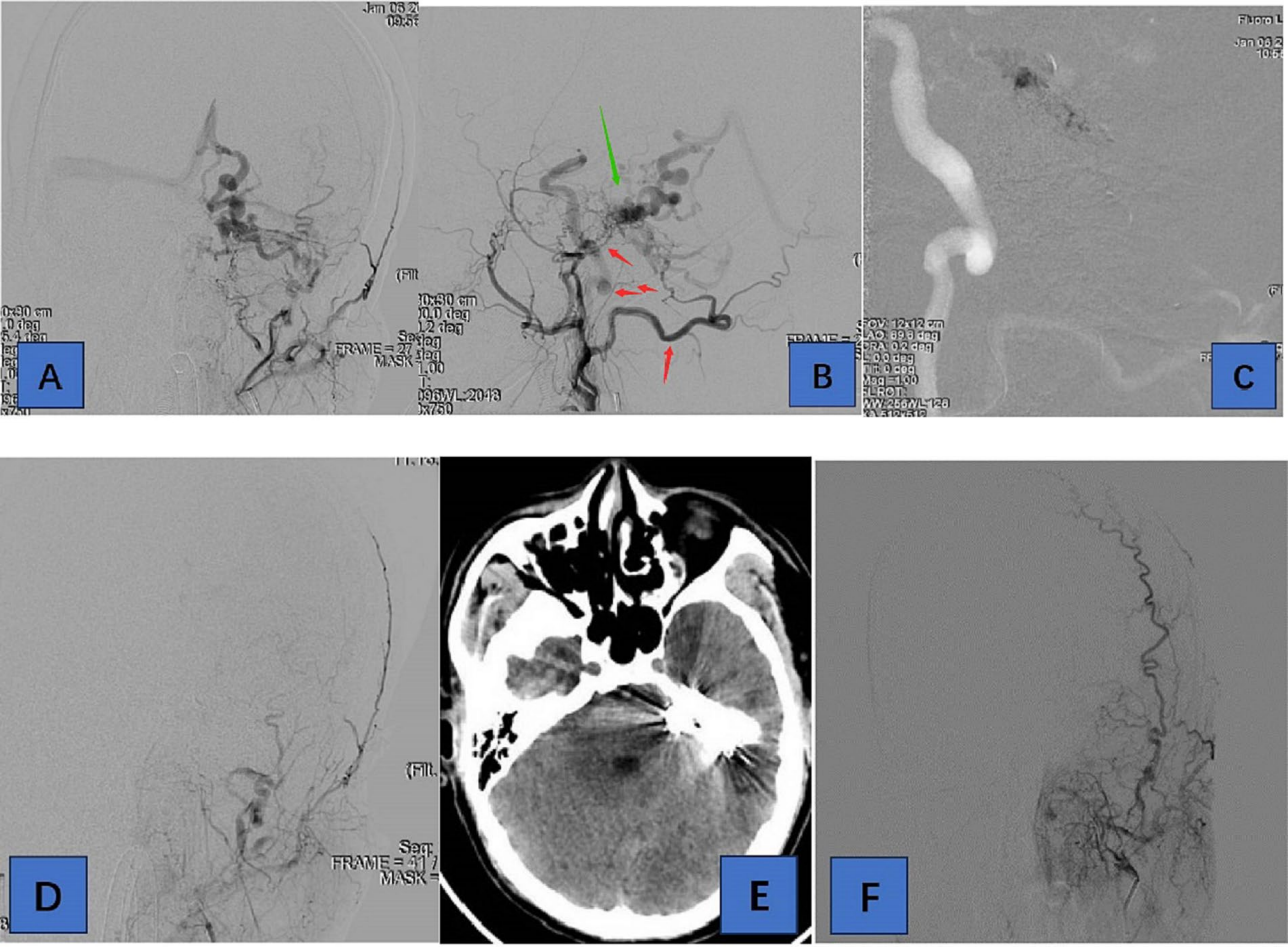
Adult patient presented with headache for 1 week, whose DSA suggested a tentorial dural arteriovenous fistula of type G6. **(A,B)** are the frontal and lateral views of the left external carotid artery, showing the left occipital, posterior auricular, middle meningeal, and ascending pharyngeal arteries involved in the blood supply. **(C)** We chose the left occipital artery route for embolization of TDAVF. **(D)** is a postoperative left external carotid arteriogram showing complete embolization of the TDAVF. **(E)** is a postoperative cranial CT showing hyperdense embolic shadow. In **(F)**, there was no recurrence of TDAVF on 3-month postoperative angiography. Red arrows point to arteries, and green arrows point to TDAVF.

The results of the present study support several larger series (6, 8, 9) that show embolization of tentorial DAVFS can be achieved with the middle meningeal artery serving as the main route of embolization. We demonstrate a 94.7% successful treatment of different types of TDAVFs, with an acceptable complication rate of 15.8%. Treatment in the present series was indicated in most cases (47.4% hemorrhage and 52.6% non-hemorrhagic symptoms) by the severity of symptoms and radiological evidence of cortical venous reflux (100%). Symptomatic TDAVF combined with cortical venous drainage has an extremely poor prognosis. Van Dijk et al. (10) reported five patients with TDAVF who were untreated or uncured, with three deaths and one case of moderate disability during follow-up. Natural regression is unfavorable even in TDAVF without combined cortical venous drainage. Therefore, TDAVF should be treated aggressively once diagnosed, even without severe clinical symptoms, and untimely treatment may be likely to lead to disease progression (Figures 1, 2).

### Aetiology of TDAVFs

Tentorial dural arteriovenous fistula (TDAVF) is an abnormal arteriovenous short circuit located at the tentorium or its accessory structures and is a relatively rare type of dural arteriovenous fistula, accounting for 4.0 to 8.4% of DAVF (11, 12). The pathogenesis of DAVF remains unclear, and most scholars believe that it is related to several acquired factors, such as head trauma, thrombosis, intracranial inflammatory response, craniotomy, and tumors (13–16). Diana et al. reported a case that iatrogenic DAVF developed on the same draining vein of a previously treated pial micro-arteriovenous malformation, which suggested that the unresected venous drainage of an AVM might be the substratum for neo-angiogenetic processes (17). A few cases of DAVF are caused by congenital factors, such as abnormal venous sinus development and genetic mutations (15, 18–20). External factors or their own factors lead to venous sinus hypertension, further causing cerebral tissue hypoperfusion and hemodynamic changes, both of which promote the expression of neovascular factors and cause abnormal neovascularization of arteries and veins, resulting in the formation of arteriovenous fistulas, which is the mainstream view of the pathogenesis of DAVF (15, 21, 22). Kojima et al. suggested that venous hypertension is associated with increased vascular endothelial growth factor (VEGF) expression, which may further induce DAVF proliferation (23). A Japanese group also supported this theory through experimental studies in rats, suggesting that a vascular endothelial growth factor may contribute to DAVF growth in patients with venous hypertension-induced ischemia (24). An in-depth study has shown that VEGF receptor antagonists reduce the induction of DAVF in rats (25).

### Rationale for treatment

DAVFs are classically defined as high risk according to the Borden and Cognard classification when cortical venous drainage is observed. This pattern of venous drainage appears to be very common in TDAVF (7, 26), making most TDAVF Borden II-III (27, 28) or Cognard IIb-IV (2, 29). Lawton et al. (7) reported that of 31 patients with TDAVF, 84% were Borden type III, and 16% were Borden type II. The 22 patients reported by Tomak et al. (30) were all Borden type III. This may explain why a malignant clinical presentation is more frequently observed in TDAVF, with 97% of patients having hemorrhagic or progressive neurological deficits (11, 31). In this study, nine patients (47.4%) presented with cerebral hemorrhage. The most common site of hemorrhage is close to the location of the fistula, including fatal hemorrhages in the posterior cranial fossa (32).

### Impact of anatomy on treatment

There are six major sources of arteries feeding the TDAVF: Bernasconi-Cassinari, middle meningeal, posterior meningeal, Davidoff-Schecter, scalp, and other branches of the external carotid artery. The origin and number of feeding arteries guide endovascular treatment, with Rezende et al. reporting that <5 feeding arteries predict complete TDAVF embolization in one session (6). In this study, there were < 5 feeding arteries in 13 patients, of whom 11 attained complete obliteration following a single endovascular treatment.

Good vascular access is a prerequisite for endovascular embolization, with arterial embolization being the first treatment choice. To confirm appropriate arterial access, the choice of vessel is based on the degree of curvature, diameter, and length of the vessel, all of which are conducive to the microcatheter tip reaching the fistula. In this study, arterial access was chosen to embolize the TDAVF in 18 patients, and only one patient was not completely embolized because of the large number of supplying arteries, involvement of important veins in drainage, and high therapeutic risk. Because most patients with TDAVF have soft meningeal veins involved in drainage, it has been suggested that venous embolization is unsuitable for the treatment. However, if arterial access is difficult to achieve and the draining vein is relatively simple in structure, venous access can be attempted (26, 29). In this study, one patient presented with a Lawton type V TDAVF and was selected for intravenous access embolization of the TDAVF (using Onyx glue and coils). The arterial approach combined with the venous approach is another strategy, but it has been reported less frequently (6).

### Implications for clinical practice

The goal of TDAVF treatment is the interruption of the fistula. The modality of treatment is affected by the complexity of arterial supply, deep location, and hemodynamic characteristics of the TDAVF. Advances in endovascular treatments have led to the primacy of this modality over open surgical management in contemporary practice. Several large series have confirmed endovascular treatment to be efficacious and safe, including the Rezende et al. (6) report of 91% (41/45pts) successful endovascular treatment of TDAVF (40 arterial access, 2 venous access, 2 combined arterial and venous access, and 1 combined arterial access with surgical resection). However, compared with dural arteriovenous fistulas at other sites, tentorial dural arteriovenous fistulas are associated with a higher risk of treatment and poorer prognosis. Li et al. (33) found a higher incidence of poor functional outcomes in the use of Onyx to treat tentorial DAVFs than for other DAVFs. There was no single treatment strategy that was suitable for all types of TDAVF, and we could only try to select an appropriate strategy that had the least risk and greatest benefit through previous treatment experience.

Over the years, various embolic agents have been used for endovascular treatment. Among them, Onyx glue is a non-adhesive liquid embolic agent suitable for treating DAVF with cortical venous drainage. In this study, all patients underwent embolization using Onyx glue. Glubran is an adhesive liquid embolization material, which is difficult and risky to use because of its disadvantages of rapid coagulation and uncontrollability and requires an experienced operator to appropriately manage the concentration configuration and determine the timing of extubation. In addition, studies have reported two new embolic agents, Squid and PHIL. The advantages of the new embolic agents over Onyx glue include ultra-low viscosity versions, more stable visibility, and a lower degree of imaging artifacts (34).

To reduce some of the complications related to Onyx, we adopt the following principles at our institution: (1) when embolization is performed from the posterior branch of the middle meningeal artery, the embolic glue should not be allowed to reflux close to the level of the foramen spinosum to avoid damaging the arterial supply of the trigeminal and facial nerve. (2) The microcatheter should be placed in or close to the fistula to ensure the Onyx glue maximizes diffusion and minimizes distal reflux. When using liquid suppositories such as Onyx gel, reflux can be controlled by repeatedly pausing the injection using the “push and push” technique (35). (3) To reduce complications, care should be taken to protect the important arteries, veins, and venous sinuses intraoperatively (e.g., using the venous sinus balloon technique). For example, when meningeal-pituitary trunk access is selected, to prevent Onyx glue from refluxing into the internal carotid artery, a balloon can be used for protection.

Route of embolization has an impact on complications and efficacy of embolization. Gioppo et al. (9) found that the MMA is commonly utilized to access cerebral DAVFs for its specific anatomical configuration, facilitating the penetration of the embolic agent to cast the DAVF. Treatment of DAVF via the arterial route is the preferred route at our center. When the middle meningeal artery is involved in blood supply, we try to use this as the main embolization route because its access is safer. The following technical aspects should be noted during embolization: precise positioning of the embolization target, including the fistula and nearest venous outlet. The transvenous route to embolization is an important route, with complications reduced by limiting occlusion to the diseased segment of the fistula whilst avoiding nearby normal cortical veins. We utilized the transvenous route in one case in the current series. In this procedure, the microcatheter was successfully over-selected to the fistula via the superior petrosal sinus route, and the fistula was filled with coils to limit excessive dispersion of Onyx glue and reduce reflux, and then Onyx glue was slowly injected to achieve complete embolism (using the technique of “reflux-retention-re-injection” to avoid complications). Unfortunately, not all TDAVFs can be occluded successfully in a single stage via either the arterial or venous route. When the tentorial dural arteriovenous fistula is complicated by many feeding arteries and important draining veins, we consider staged endovascular treatment until complete embolization of the TDAVF. We chose this strategy for four patients in the current report, with a 3-month interval between stages. Prolonging the interval risks provoking a bleed from a change in the flow dynamics through the fistula. Finally, when complex DAVFs present with no safe single modality option for cure, we recommend combined open and endovascular surgery. The therapeutic goal for one such case in our series was to reduce the risk of cerebral hemorrhage using flow reduction surgery.

### Limitations

The case series was from a single center with a small sample size, and the potential risks and complications of endovascular treatment of TDAVF may be different if a larger population were included.

## Conclusion

Endovascular treatment for tentorial dural arteriovenous fistulas is safe and effective. Where complete obliteration of the fistula is unattainable, lowering the Borden or Cognard grade through the elimination of cortical venous drainage should be aimed for. Large studies are required to validate this management approach.

## Data availability statement

The original contributions presented in the study are included in the article/supplementary material, further inquiries can be directed to the corresponding authors.

## Ethics statement

Ethical review and approval was not required for the study on human participants in accordance with the local legislation and institutional requirements. Written informed consent from the patients/participants or patients/participants’ legal guardian/next of kin was not required to participate in this study in accordance with the national legislation and the institutional requirements.

## Author contributions

GZ: Writing – original draft, Writing – review & editing. WZ: Investigation, Writing – review & editing. HC: Investigation, Writing – review & editing. YS: Investigation, Software, Writing – review & editing. CM: Data curation, Methodology, Writing – review & editing. LM: Data curation, Supervision, Writing – review & editing. ZL: Investigation, Resources, Supervision, Writing – review & editing. HL: Funding acquisition, Resources, Visualization, Writing – review & editing.
